# 

**Published:** 1881-08

**Authors:** 


### CONTENTS:

ART. I.—Alveolar Ulceration as Distinguished from Alveolar Abscess.
By L. C Ingersoll, D D. S....145
ART. II.—Proceedings of the Mississippi State Dental Association....154
ART. III.—Rapid Breathing as a Pain Obtunder, etc , (Continued.)
By W. G. A. Bonwill, D. D. S....163
ART. IV.—Restoring Partial Loss of Teeth without Plate....175
ART. V.—Mode of Origin of Some Secondary Lesions.
By Ed. W. Cox, L. D S , R. C. L. S....180
Meeting of Maryland and District of Columbia Dental Society....185

### EDITORIAL, ETC.

A New Source of Electricity....186
The Stomatoscope....187
Omission....188

### OBITUARY.

Byron F. Coy, D. D. S....188

### MONTHLY SUMMARY.

Sensitive Dentine....190
To Prevent Clouding of Mirrors by Moisture....190
Dangers of Dentistry....191
Analyzing Metals by Electrolysis....192
Dangers of Bonwill Method....192

